# Emotional and Decisional Profiles in Individuals With Gambling Disorder: Associations With Emotional Dysregulation, Alexithymia, and Suicidal Ideation

**DOI:** 10.62641/aep.2380

**Published:** 2026-10-15

**Authors:** Sara Meca, Maragda Puigcerver, Noemí SanMiguel, Miguel-Angel Serrano

**Affiliations:** ^1^Department of Psychobiology, University of Valencia, 46010 Valencia, Spain

**Keywords:** Gambling Disorder, emotional dysregulation, alexithymia, decision-making, suicidal ideation

## Abstract

**Background:**

Gambling Disorder (GD) is associated with elevated emotional dysregulation, maladaptive decision-making, and heightened suicide risk, yet the relationships among these domains remain insufficiently integrated within a single clinical framework.

**Methods:**

Forty-four treatment-seeking adults with a Diagnostic and Statistical Manual of Mental Disorders (5th edn, DSM-5) diagnosis of GD completed measures of gambling severity (National Opinion Research Center DSM-IV Screen for Gambling Problems (NODS)), emotional dysregulation (Difficulties in Emotion Regulation Scale (DERS)), alexithymia (20-item Toronto Alexithymia Scale (TAS-20)), hot decision-making (Iowa Gambling Task (IGT), Mixed Gamble Task (MGT)), cold decision-making and decisional style (Lottery Choice Task (LCT), General Decision-Making Style questionnaire (GDMS)), suicidal ideation (Columbia-Suicide Severity Rating Scale (C-SSRS)), and psychological functioning (Symptom Checklist-90-Revised (SCL-90-R), Satisfaction With Life Scale (SWLS)). Exploratory correlational analyses and k-means clustering, confirmed with hierarchical clustering, were conducted.

**Results:**

Higher emotional dysregulation was associated with lower loss aversion in hot decision-making and with more avoidant and spontaneous decision-making styles; recent gambling severity was also associated with lower loss aversion as well. Higher alexithymia was associated with reduced gain and loss sensitivity in cold decision-making. Cluster analysis identified a higher-risk profile characterized by greater emotional dysregulation, higher recent gambling severity, greater suicidal ideation, higher alexithymia, and a more spontaneous decisional style.

**Conclusions:**

Although exploratory, these findings support a multidimensional risk framework in which emotional vulnerability and decisional tendencies jointly characterize clinically relevant heterogeneity in GD. The results underscore the importance of incorporating emotion-focused and suicide-risk assessment strategies into the clinical evaluation of individuals with GD.

## Introduction

Gambling Disorder (GD) is recognized in the Diagnostic and Statistical Manual of Mental Disorders (5th edn; DSM-5) as a behavioral addiction characterized by persistent and recurrent gambling behavior associated with clinically significant impairment [1]. As in substance-related addictions, GD is commonly accompanied by affective distress, impulsivity, and impaired self-regulation, and it is increasingly conceptualized as a disorder in which cognitive, emotional, and motivational processes interact to maintain maladaptive behavior [2, 3].

Suicide risk is one of the most clinically important correlates of GD. Meta-analytic evidence indicates markedly elevated rates of suicidal ideation and suicide attempts among individuals with gambling problems relative to the general population, while registry-based studies suggest substantially increased suicide mortality in diagnosed GD samples [2, 4, 5, 6]. Indeed, both an earlier Swedish cohort and a recent Norwegian cohort identify suicide as the leading cause of death in diagnosed GD, accounting for roughly a quarter to a third of all deaths [5, 7]. Although psychiatric comorbidity, particularly depression, contributes to this risk, GD-specific factors such as financial strain, shame, interpersonal deterioration, and loss of perceived control may also increase vulnerability to suicidal thinking and behavior.

Within this context, emotional dysregulation has emerged as a particularly relevant transdiagnostic mechanism. Emotional dysregulation encompasses difficulties in identifying, accepting, modulating, and behaviorally managing emotional states [8]. In GD, higher levels of emotional dysregulation have been linked to greater symptom severity, maladaptive coping, and more disadvantageous decision-making [9]. This construct is clinically important because gambling may function, at least for a subgroup of individuals, as a maladaptive strategy for reducing aversive internal states rather than simply as reward seeking.

Alexithymia may further intensify this vulnerability. Defined by difficulties identifying and describing feelings together with a tendency toward externally oriented thinking [10], alexithymia has repeatedly been associated with GD and with broader markers of emotional maladjustment, including hopelessness and suicidality [11, 12]. Individuals with alexithymic traits may be less able to recognize distress signals, communicate emotional need, and use adaptive regulatory strategies, thereby increasing reliance on gambling as a dysfunctional form of emotional management.

Decision-making may represent one pathway through which emotional vulnerability becomes behaviorally expressed. Research in GD distinguishes between “hot” decision-making, which involves affectively charged reward and loss contingencies, and “cold” decision-making, which relies more on deliberative evaluation and stable decisional style. Both domains appear altered in GD [13]. Moreover, alexithymia has been linked to poorer performance in ambiguity-based tasks such as the Iowa Gambling Task (IGT), suggesting that emotional processing deficits may compromise the use of feedback during choice behavior [14]. From this perspective, emotional dysregulation and maladaptive decision-making are better understood as interacting rather than independent vulnerabilities. Although these domains have largely been studied in isolation—emotional dysregulation and decision-making [9], alexithymia and decision-making [14], and emotional dysregulation or alexithymia and suicidality [2, 11]—they are theoretically interdependent and plausibly co-occur within the same individuals [3, 13], yet they have rarely been integrated within a single clinical sample. Considering the emotional (emotional dysregulation, alexithymia) and clinical (gambling severity, suicidal ideation) domains together, as they are expressed in the decision-making process, may therefore capture this complexity better than studying each in isolation. The present study therefore had two aims: first, to examine associations between emotional variables and decision-making parameters; and second, to explore whether clinically meaningful profiles could be identified on the basis of emotional dysregulation, suicidal ideation, and recent gambling severity, and whether those profiles differed in alexithymia, decisional style, and psychological functioning. We expected higher emotional dysregulation and alexithymia to be associated with greater gambling severity, higher suicide risk, and less adaptive decisional functioning. Given the modest sample size and the limited prior literature integrating these variables, analyses involving specific decisional parameters and profile comparisons were treated as exploratory.

## Materials and Methods

### Participants

Participants were 44 adults with a DSM-5 diagnosis of GD [1], recruited from specialized behavioral-addiction services in Spain between January 2022 and December 2023. In each case the diagnosis was established by a licensed clinical psychologist as part of routine clinical care, through a semi-structured clinical interview based on the DSM-5 criteria. Gambling severity was subsequently quantified with the National Opinion Research Center DSM-IV Screen for Gambling Problems (NODS), a structured screen indexing the Diagnostic and Statistical Manual of Mental Disorders, Fourth Edition (DSM-IV) criteria for pathological gambling (see Instruments). Because the NODS predates the DSM-5, it was used to characterize gambling severity rather than to establish the diagnosis, which followed DSM-5 criteria; eligible patients were recruited consecutively over the study period, and those who met the diagnostic threshold were invited to participate. Exclusion criteria were the presence of dual pathology, i.e., a co-occurring psychiatric or substance-use disorder other than GD, as recorded in the patient’s clinical history at the specialized service. Of 49 patients assessed for eligibility, 5 were excluded on this basis, and all 44 eligible patients agreed to participate; there were no refusals. The participant flow (screening, enrolment, and the analytic sample) is summarized in the Strengthening the Reporting of Observational Studies in Epidemiology (STROBE)-style participant-flow diagram (Fig. 1, Ref. [15]).

**Fig. 1.**
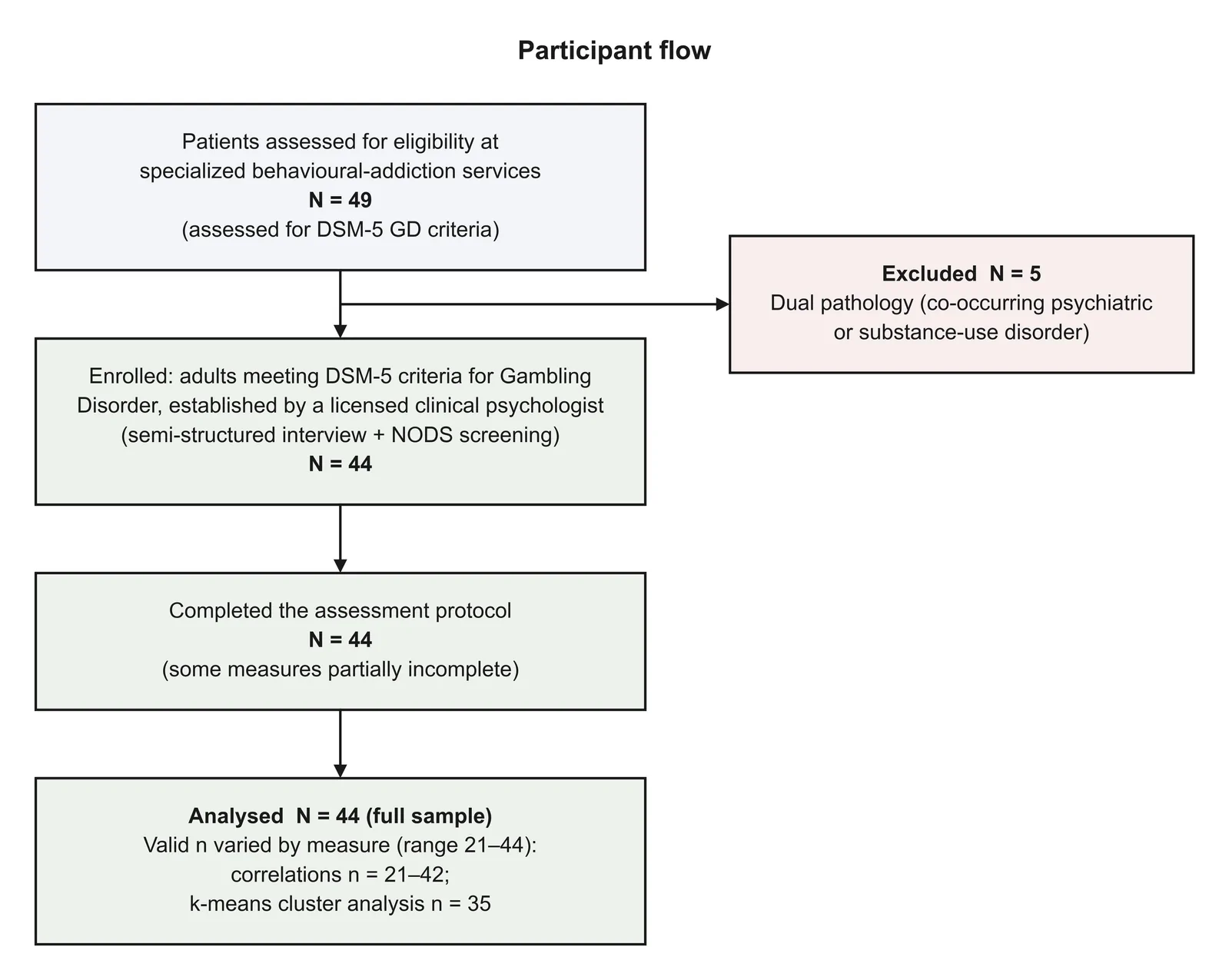
**STROBE-style participant-flow diagram for the study sample [15]**. DSM-5, Diagnostic and Statistical Manual of Mental Disorders (5th edn); GD, Gambling Disorder; NODS, National Opinion Research Center DSM-IV Screen for Gambling Problems. The diagram shows screening (n = 49 assessed, 5 excluded for dual pathology), enrolment (n = 44), completion, and the analytic sample (valid n ranging from 21 to 44 across analyses).

Because some participants did not complete all questionnaires or tasks, valid sample sizes varied across analyses, ranging from n = 21 to n = 44 (full sample); the exact n is reported for every analysis and in each table.

### Procedure and Design

This study employed a cross-sectional observational design; each participant was assessed at a single time point, with no longitudinal follow-up. After diagnostic confirmation, eligible patients were informed about the aims and procedures of the study and invited to participate voluntarily. Assessment was conducted across several sessions, depending on each participant’s availability, clinical status, and pace of completion, in order to reduce burden and facilitate completion of the full protocol.

### Instruments

Gambling severity was assessed using the NODS [16], a structured interview-based instrument developed to reflect the DSM-IV diagnostic criteria for pathological gambling and widely used in both clinical and research settings. The NODS consists of 17 items that map onto the 10 DSM-IV diagnostic categories for pathological gambling; each item block is administered for two timeframes—lifetime and past-year—yielding 34 items in total; for each timeframe the number of criteria met is scored from 0 to 10. A timeframe score of 1–2 is labeled “at risk”, 3–4 a “problem gambler”, and 5–10 a “pathological gambler” [16]. In the present study, both lifetime and past-year indices were computed in order to capture overall gambling history as well as more recent symptom severity. Sample eligibility was defined by a clinical DSM-5 diagnosis of GD rather than by any NODS cutoff; NODS scores were used dimensionally (continuous severity) in all inferential analyses, and the categorical NODS bands (at risk, problem, and pathological gambler) are reported for descriptive purposes only (Table 1). The instrument has shown good reliability and validity in clinical and epidemiological studies [16]. In the present sample, internal consistency of the criteria-level past-year index was excellent (α = 0.92); the lifetime index was less internally consistent (α = 0.62), attenuated by a ceiling effect, as almost all participants (38 of 40 with valid lifetime scores, 95.0%) met the pathological-gambler threshold (≥5 criteria).

**Table 1.** **Demographic and clinical characteristics of the sample (n = 44)**.

Variable		Values
Age (years), M (SD) [range]		36.6 (11.6) [18–61]
Gender—men, n (%)		41 (93.2)
Marital status, n (%)		
	Single	23 (52.3)
	Married	13 (29.5)
	Partnered	3 (6.8)
	Divorced	3 (6.8)
	Missing	2 (4.5)
Educational level, n (%)		
	Primary/Compulsory	10 (22.7)
	Secondary/Vocational	23 (52.3)
	University	10 (22.7)
	Missing	1 (2.3)
Employment status, n (%)		
	Employed	27 (61.4)
	Unemployed	13 (29.5)
	On sick leave	1 (2.3)
	Student	2 (4.5)
	Missing	1 (2.3)
On medication at assessment, n (%)		
	Yes	14 (31.8)
	No	29 (65.9)
	Missing	1 (2.3)
Lifetime gambling severity (NODS), n (%)		
	Pathological (≥5 criteria)	38 (86.4)
	Problem (3–4 criteria)	2 (4.5)
	Missing	4 (9.1)
Current suicidal ideation (C-SSRS ≥ 1), n (%)		
	Present	11 (25.0)
	Absent	32 (72.7)
	Missing	1 (2.3)

M, mean; SD, standard deviation; NODS, National Opinion Research Center DSM-IV Screen for Gambling Problems; C-SSRS, Columbia-Suicide Severity Rating Scale. Lifetime gambling-severity categories were derived from the 40 participants with a valid NODS lifetime score (4 missing); the percentages shown for these categories are computed on n = 44, whereas among valid cases 38 of 40 (95.0%) met the pathological threshold (≥5 criteria) and 2 of 40 (5.0%) met the problem-gambling threshold (3–4 criteria). Unless otherwise indicated, percentages are based on n = 44 and may not total 100 due to rounding.

Emotional dysregulation was assessed with the 28-item Spanish version of the Difficulties in Emotion Regulation Scale (DERS), a self-report measure assessing five domains of the Spanish factor structure: emotional rejection (negative secondary reactions to one’s own distress), lack of emotional control, daily interference (difficulty with goal-directed behavior when distressed), emotional inattention (lack of awareness of emotional states), and emotional confusion (lack of emotional clarity) [8]. Items are rated on a 5-point scale; five items are reverse-scored, and higher scores indicate greater difficulties in emotion regulation. In the present sample, internal consistency was excellent for the DERS total (α = 0.95) and good to excellent for all subscales (emotional rejection α = 0.85, lack of emotional control α = 0.91, daily interference α = 0.87, emotional inattention α = 0.84, emotional confusion α = 0.90).

Alexithymia was assessed using the 20-item Toronto Alexithymia Scale (TAS-20) [10], one of the most widely used self-report instruments for the assessment of alexithymic traits. Participants rated each item on a 5-point Likert scale ranging from 1 (strongly disagree) to 5 (strongly agree). Total scores range from 20 to 100, with higher scores indicating greater alexithymic characteristics. The validated Spanish version of the TAS-20 was administered [17]. The instrument assesses three dimensions: Difficulty Identifying Feelings (DIF), Difficulty Describing Feelings (DDF), and Externally Oriented Thinking (EOT). In the present sample, internal consistency was acceptable for DIF (α = 0.82) and EOT (α = 0.69) but poor for DDF (α = 0.46); accordingly, DDF-based results are reported for completeness but interpreted with caution throughout, as its low reliability attenuates and destabilizes any association involving this subscale.

Decision-making was assessed through both hot and cold decision-making measures. Hot decision-making was evaluated using two behavioral paradigms. First, participants completed the IGT [18, 19], a computerized task assessing decision-making under ambiguity through trial-and-error learning. Participants select cards from four decks across 100 trials, with two decks being disadvantageous in the long term and two being advantageous. Performance is indexed by a net score computed as advantageous minus disadvantageous choices; lower scores indicate riskier decision-making.

Hot decision-making was also assessed with the Mixed Gamble Task (MGT) [20, 21], a paradigm evaluating choices under risk and loss aversion. Participants repeatedly decide whether to accept or reject mixed gambles involving potential gains and losses of varying magnitude. The task yields computational parameters such as risk sensitivity (rho), loss aversion (lambda), and choice consistency (tau).

Cold decision-making was assessed using the Lottery Choice Task (LCT) [22] and the General Decision-Making Style questionnaire (GDMS) [23, 24]. The LCT presents participants with explicit information about outcome probabilities and magnitudes, minimizing ambiguity and providing indices of gain sensitivity and loss sensitivity under relatively controlled and cognitively mediated conditions.

We used the validated Spanish version of the GDMS [24]. The original English instrument [23] comprises 25 items, five for each of the five decisional styles. The Spanish adaptation removed one Spontaneous item that cross-loads consistently with the Intuitive scale and, following confirmatory factor analysis, two Intuitive items with factor loadings below 0.30, yielding a final 22-item version in which the Rational, Dependent and Avoidant styles retain 5 items each, the Spontaneous style comprises 4 items, and the Intuitive style comprises 3 items [24]. In the present study, only the Rational, Spontaneous, and Avoidant styles were considered in the analyses, focusing on those decisional tendencies most closely related to gambling-related behavior and clinical risk. Responses are rated on a 5-point Likert scale and summed within each subscale, so scores range from 5 to 25 (Rational, Avoidant) and from 4 to 20 (Spontaneous) [24]. In the present sample, internal consistency (Cronbach’s α) was 0.66 for GDMS Rational, 0.91 for Spontaneous, and 0.94 for Avoidant; the corresponding values in the Spanish validation study were 0.72, 0.87 and 0.91, with eight-week test-retest intraclass correlations of 0.77, 0.81 and 0.86 [24].

Suicidal ideation and suicidal behavior were assessed using the Columbia-Suicide Severity Rating Scale (C-SSRS) [25, 26], a standardized clinician-administered instrument. The C-SSRS assesses suicidal ideation along five levels of increasing severity, ranging from a passive wish to be dead to the presence of a specific suicidal plan with intent. For the exploratory clustering analyses, suicidal ideation was dichotomized as any current ideation (C-SSRS ideation severity ≥1, i.e., levels 1–5) versus none (level 0). This split is definitional rather than an empirically derived cutoff: level 0 corresponds to the absence of ideation, so any positive level indicates present ideation [25, 26]. The C-SSRS has established convergent and predictive validity for suicidal ideation and behaviour, and the Spanish version has shown adequate psychometric properties [26]; as a clinician-administered interview in which ideation is captured by a single severity rating rather than by a set of parallel items, an internal-consistency coefficient is not applicable.

General psychopathological symptoms were assessed with the Symptom Checklist-90-Revised (SCL-90-R) [27], a 90-item self-report inventory in which each item is rated on a 5-point scale from 0 to 4. As indicators of general psychological distress and affective symptomatology, we used the Global Severity Index (GSI), computed as the mean of all 90 items, together with the depression (13 items) and anxiety (10 items) subscales, each scored as the mean of its constituent items; all three indices therefore range from 0 to 4, with higher scores indicating greater symptom severity. The SCL-90-R and its Spanish adaptation have well-established internal consistency and validity across clinical and community samples [27].

Subjective well-being was assessed with the Satisfaction With Life Scale (SWLS) [28], a brief 5-item self-report measure evaluating individuals’ global judgment of their life satisfaction, rated on a 7-point Likert scale from 1 (strongly disagree) to 7 (strongly agree). Total scores are obtained by summing the five items and range from 5 to 35, with higher scores indicating greater life satisfaction. In the present sample, Cronbach’s α was 0.81.

### Data Analysis

Descriptive statistics were computed for all study variables, and the distribution of each quantitative variable was examined with the Shapiro–Wilk test. Most scale totals were consistent with normality, whereas recent and lifetime gambling severity (NODS), the SCL-90-R Global Severity and anxiety indices, and the behavioral decision-making indices (IGT and LCT) departed from normality; for these, Spearman correlations and Mann–Whitney tests are reported as nonparametric checks.

Pearson product-moment correlations were used to examine bivariate associations between emotional regulation variables (DERS total score and TAS-20 total/subscales) and decision-making indices (MGT loss aversion parameter, and GDMS decision-style subscales). Given the number of correlations computed and the presence of some non-normal variables, three robustness procedures were added: 95% confidence intervals (CIs), Spearman rank correlations, and Benjamini–Hochberg false-discovery-rate (FDR) correction. Statistical significance was set at α = 0.05, and effect sizes were interpreted according to Cohen’s [29] conventions.

An exploratory k-means cluster analysis was performed to identify subgroups within the sample based on standardized scores for overall emotion dysregulation (DERS total), suicidal ideation (coded 0/1), and recent gambling severity (NODS past-year score). Standardizing the inputs prevents the widest-ranging variable (DERS) from dominating the Euclidean distance. Of the 44 participants, 35 had complete data on all three clustering variables and were included. The two-cluster solution was chosen a priori for clinical interpretability and evaluated with the silhouette coefficient and with bootstrap resampling; its robustness was further assessed against an unstandardized solution and against an agglomerative hierarchical (Ward) clustering of the same standardized variables. Between-cluster differences on continuous variables were tested with one-way Analysis of Variances (ANOVAs) (with Mann–Whitney U as a nonparametric check), and the dichotomous suicidal-ideation variable was compared with Fisher’s exact test rather than being treated as continuous.

All analyses were conducted in Python v3.9.6 (Python Software Foundation, Beaverton, OR, USA), using the open-source libraries pandas v2.3.3, NumPy v2.0.2, SciPy v1.13.1, scikit-learn v1.6.1, and Matplotlib v3.9.4 (NumFOCUS Inc., Austin, TX, USA), and statsmodels v0.14.6.

## Results

### Descriptive Statistics

The demographic and clinical characteristics of the 44 participants are summarized in Table 1.

Descriptive statistics for key study variables are shown in Table 2. Overall, the sample showed elevated emotional-regulation difficulties, particularly in lack of emotional control, emotional rejection, and daily interference with goal-directed behavior when distressed. Alexithymia scores were driven primarily by difficulty identifying feelings and externally oriented thinking. With regard to decisional style, participants reported higher rational decision-making than avoidant or spontaneous styles. Life satisfaction was low, psychological distress was moderate, and depressive symptoms were somewhat higher than anxiety symptoms. Recent gambling severity showed substantial variability across participants, indicating notable clinical heterogeneity within the sample.

**Table 2.** **Means, standard deviations (SDs), ranges, and valid sample sizes for the key study variables**.

Variable	M	SD	Range	n
DERS total	69.09	23.36	28–121	43
Emotional rejection	17.23	6.58	7–29	43
Lack of emotional control	20.85	8.75	9–45	43
Daily interference	11.63	4.40	4–20	43
Emotional inattention	10.02	3.59	4–16	43
Emotional confusion	9.35	4.14	4–20	43
TAS-20 total	51.56	11.51	28–78	43
DIF	16.28	6.76	7–33	43
DDF	14.33	4.25	5–21	43
EOT	20.95	3.62	14–30	43
GDMS				
GDMS Rational	17.69	3.05	11–25	42
GDMS Avoidant	13.83	5.70	5–25	42
GDMS Spontaneous	11.14	4.15	4–20	42
Decision-making tasks				
IGT Total	–1.19	25.03	–60–60	42
MGT loss aversion (λ)	1.53	0.37	0.70–2.17	24
LCT gain sensitivity	2.99	2.19	0.85–6.00	40
LCT loss sensitivity	3.52	2.34	0.00–6.00	40
SWLS	13.61	4.41	5–24	41
SCL-90-R GSI	1.17	0.88	0.00–3.75	37
Depression subscale	1.37	0.98	0.00–3.46	37
Anxiety subscale	1.07	0.99	0.00–3.50	37
NODS				
Past year	5.11	3.82	0–10	37
Lifetime	8.38	1.71	3–10	40

M, mean; SD, standard deviation; DERS, Difficulties in Emotion Regulation Scale; TAS-20, 20-item Toronto Alexithymia Scale; DIF, Difficulty Identifying Feelings; DDF, Difficulty Describing Feelings; EOT, Externally Oriented Thinking; GDMS, General Decision-Making Style questionnaire (subscales are summed scores: Rational/Avoidant 5–25, spontaneous 4–20); IGT, Iowa Gambling Task (net score, advantageous minus disadvantageous choices); MGT, Mixed Gamble Task (λ = loss-aversion parameter); LCT, Lottery Choice Task; SWLS, Satisfaction With Life Scale; SCL-90-R, Symptom Checklist-90-Revised; GSI, Global Severity Index; NODS, National Opinion Research Center DSM-IV Screen for Gambling Problems (values reflect the number of DSM-IV criteria met, 0–10). n = valid sample size for that variable, valid n varies across variables because of incomplete questionnaires or tasks.

### Exploratory Correlational Analyses

In the hot decision-making domain, DERS total scores were negatively correlated with the MGT loss-aversion parameter (r = –0.53, 95% CI [–0.77, –0.17], n = 24, *p* = 0.007; Spearman ρ = –0.52), indicating that greater emotional dysregulation was associated with reduced sensitivity to losses during reward-laden choice.

In the cold decision-making domain, TAS-20 total scores were negatively correlated with both LCT gain sensitivity (r = –0.54, 95% CI [–0.73, –0.28], n = 40, *p* < 0.001) and LCT loss sensitivity (r = –0.36, 95% CI [–0.60, –0.05], n = 40, *p* = 0.023), suggesting that higher alexithymia was associated with reduced differentiation of outcome valence under more deliberative choice conditions. This pattern was not specific to alexithymia, however: LCT gain sensitivity was also negatively correlated with DERS total scores (r = –0.45, 95% CI [–0.67, –0.16], n = 39, *p* = 0.004) and with recent gambling severity (r = –0.53, 95% CI [–0.73, –0.23], n = 34, *p* = 0.001); LCT loss sensitivity showed a similar but weaker pattern (DERS: r = –0.29, 95% CI [–0.56, 0.02], n = 39, *p* = 0.069, not significant; NODS past-year: r = –0.39, 95% CI [–0.65, –0.06], n = 34, *p* = 0.022). Reduced sensitivity to gains on the LCT therefore tracks emotional dysregulation and gambling severity as well as alexithymia.

Regarding decision-making style, DERS total scores were positively associated with avoidant (r = 0.43, 95% CI [0.14, 0.65], n = 41, *p* = 0.005) and spontaneous (r = 0.35, 95% CI [0.05, 0.59], n = 41, *p* = 0.023) decisional styles, indicating that greater emotional dysregulation tended to co-occur with more evasive and impulsive approaches to choice. The negative association with rational decision-making did not reach significance (r = –0.28, n = 41, *p* = 0.072) and did not survive FDR correction; it is therefore not interpreted as reliable.

Additional clinically relevant associations emerged with gambling severity. DERS total scores were positively correlated with NODS past-year severity (r = 0.54, 95% CI [0.25, 0.74], n = 36, *p* < 0.001), and TAS-20 total scores were also positively associated with recent gambling severity (r = 0.40, 95% CI [0.08, 0.64], n = 36, *p* = 0.016). TAS-20 total scores were further correlated with avoidant decision-making style (r = 0.40, 95% CI [0.11, 0.63], n = 42, *p* = 0.008). Recent gambling severity was itself negatively correlated with the MGT loss-aversion parameter (r = –0.50, 95% CI [–0.77, –0.09], n = 21, *p* = 0.021), indicating that more severe recent gambling co-occurred with reduced sensitivity to losses. All of these associations, together with the two cold-decision-making correlations and the DERS–avoidant/spontaneous associations, remained significant after FDR correction. The full correlation matrix is shown in Fig. 2.

**Fig. 2.**
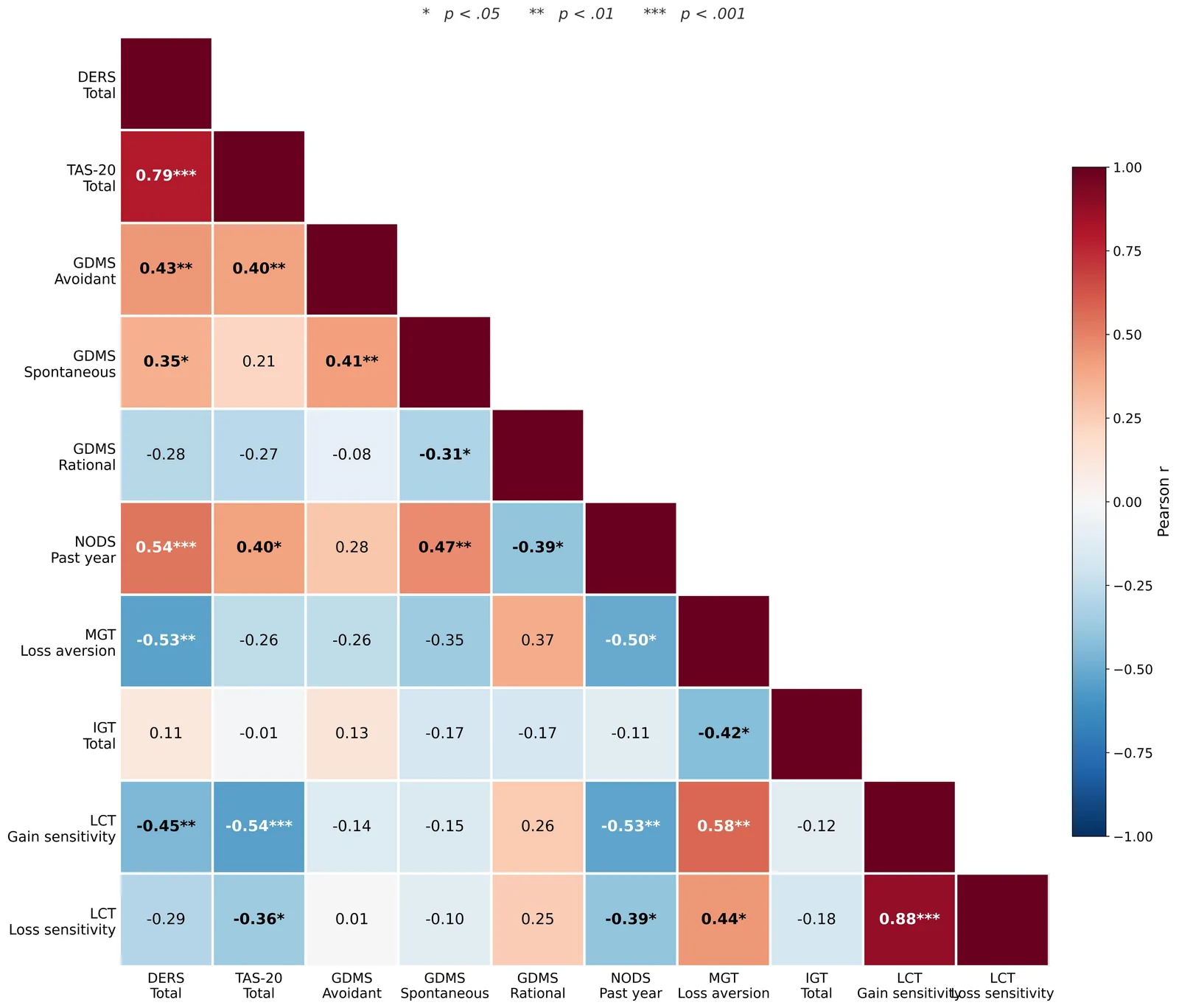
**Correlation matrix of emotional-regulation, decision-making, and clinical severity indices**. DERS, Difficulties in Emotion Regulation Scale; TAS-20, 20-item Toronto Alexithymia Scale; GDMS, General Decision-Making Style questionnaire; NODS, National Opinion Research Center DSM-IV Screen for Gambling Problems; MGT, Mixed Gamble Task; IGT, Iowa Gambling Task; LCT, Lottery Choice Task. Cells report Pearson’s r; asterisks denote significance based on uncorrected *p*-values (* *p* < 0.05, ** *p* < 0.01, *** *p* < 0.001).

### Exploratory Cluster Analyses

A two-cluster solution provided an interpretable partition of the 35 participants with complete clustering data (silhouette coefficient = 0.44). The partition was reasonably reproducible under bootstrap resampling (mean adjusted Rand index = 0.73) and agreed substantially with an agglomerative hierarchical (Ward) clustering of the same variables (adjusted Rand index = 0.68). As expected for a small sample, it retained some sensitivity to analytic choices: an unstandardized solution produced a comparable but not identical split (14 vs. 21). The solution is therefore described as exploratory and clinically oriented rather than definitive.

Cluster 1 (High emotional risk; n = 13) was characterized by higher emotional dysregulation, substantially higher prevalence of current suicidal ideation (10/13, 76.9%), and more severe recent gambling behavior. This cluster also showed higher alexithymia and more spontaneous decision-making than Cluster 2.

Cluster 2 (Lower emotional risk; n = 22) showed lower emotional dysregulation, no current suicidal ideation (0/22, 0.0%; Fisher’s exact *p* < 0.001 for the between-cluster difference), and lower recent gambling severity, with comparatively better-preserved life satisfaction.

Beyond the defining variables, the high-risk profile showed significantly higher TAS-20 total scores—especially difficulty identifying feelings—a more spontaneous decisional style, and lower life satisfaction. At the subscale level, four of the five DERS dimensions differentiated the profiles, whereas emotional inattention did not reach significance. Notably, although overall psychological distress (SCL-90-R GSI) did not differ between profiles, the depression subscale specifically did, suggesting that the high-risk profile is characterized by elevated depressive symptoms rather than generalized distress. By contrast, difficulty describing feelings, externally oriented thinking, avoidant and rational decision-making styles, overall psychological distress (SCL-90-R GSI), and anxiety symptoms did not significantly distinguish the two clusters. This pattern of significant and null contrasts was consistent across the parametric ANOVAs and the Mann–Whitney U checks. Complete descriptive statistics, F values, and effect sizes are presented in Table 3.

**Table 3.** **Between-cluster comparisons on key study variables (One-Way ANOVAs)**.

Variable		Cluster 1—High emotional risk (n = 13)		Cluster 2—Low emotional risk (n = 22)		n (H/L)	F	*p*	η ^2^
		M	SD	M	SD				
Emotional dysregulation									
	DERS total	88.6	22.89	56.82	13.82	13/22	26.43	<0.001	0.44
	Emotional rejection	23.08	4.65	13.64	4.98	13/22	30.81	<0.001	0.48
	Lack of emotional control	27.75	9.43	16.73	5.12	13/22	20.25	<0.001	0.38
	Daily interference	14.15	3.98	9.68	3.48	13/22	12.13	0.001	0.27
	Emotional inattention	11.62	3.82	9.18	3.45	13/22	3.76	0.061	0.10
	Emotional confusion	12.00	4.49	7.59	2.65	13/22	13.46	0.001	0.29
Alexithymia									
	TAS-20 total	57.46	12.57	47.10	8.99	13/21	7.86	0.009	0.20
	DIF	20.46	6.16	13.14	4.40	13/21	16.35	<0.001	0.34
	DDF	14.62	4.27	13.43	4.17	13/21	0.64	0.430	0.02
	EOT	22.38	3.95	20.52	2.87	13/21	2.53	0.122	0.07
Decision-making style									
	GDMS Avoidant	15.77	4.95	12.75	6.47	13/20	2.04	0.163	0.06
	GDMS Spontaneous	13.62	4.37	9.70	3.67	13/20	7.72	0.009	0.20
	GDMS Rational	16.38	2.90	18.4	3.25	13/20	3.29	0.080	0.10
Functioning & Gambling severity									
	NODS past-year (0–10)	7.92	2.6	3.77	3.60	13/22	13.17	0.001	0.29
	SWLS (life satisfaction)	11.23	4.02	15.00	4.00	13/20	6.96	0.013	0.18
	SCL-90-R GSI	1.46	0.79	0.94	0.94	11/20	2.40	0.132	0.08
	SCL-90-R anxiety	1.41	0.94	0.76	0.89	11/20	3.58	0.068	0.11
	SCL-90-R depression	2.02	0.92	0.94	0.78	11/20	11.91	0.002	0.29

Cluster 1—High emotional risk (n = 13); Cluster 2—Low emotional risk (n = 22); n (H/L) = valid n in C1 (High) and C2 (Low) for that row; F = one-way ANOVA F statistic (df = 1, nH + nL – 2); *p* = significance of the F test; η^2^ = eta-squared (effect size). M, mean; SD, standard deviation; DERS, Difficulties in Emotion Regulation Scale; TAS-20, 20-item Toronto Alexithymia Scale; DIF, Difficulty Identifying Feelings; DDF, Difficulty Describing Feelings; EOT, Externally Oriented Thinking; GDMS, General Decision-Making Style questionnaire; NODS, National Opinion Research Center DSM-IV Screen for Gambling Problems; SWLS, Satisfaction With Life Scale; SCL-90-R, Symptom Checklist-90-Revised; GSI, Global Severity Index. Cluster membership was derived from standardized scores of DERS total, NODS past-year (0–10), and suicidal ideation (0/1); total n = 35. The dichotomous suicidal-ideation variable was compared with Fisher’s exact test (10/13 vs. 0/22 with current ideation; *p* < 0.001) and is therefore not shown in this table. All contrasts were confirmed with Mann–Whitney U tests for non-normal variables, with unchanged conclusions.

## Discussion

This study examined whether emotional dysregulation, alexithymia, decision-making, and suicidal ideation converge in clinically meaningful ways in GD. Three findings deserve emphasis. First, greater emotional dysregulation was associated with lower loss aversion in hot decision-making and with more maladaptive decisional styles; recent gambling severity was associated with lower loss aversion as well. Second, alexithymia was associated with reduced gain and loss sensitivity in cold decision-making. Third, the exploratory cluster analysis identified a higher-risk profile characterized not only by elevated emotional dysregulation, suicidal ideation, and recent gambling severity, but also by higher alexithymia and a more spontaneous decision-making style.

The association between emotional dysregulation and reduced loss aversion is clinically meaningful because it links affective vulnerability to a decisional pattern directly relevant to gambling escalation. Individuals who struggle to regulate emotional states may be less responsive to aversive feedback during risky choice, which could weaken the inhibitory value of losses and facilitate persistence in gambling despite negative consequences. This interpretation is compatible with the somatic marker account of decision-making [30] and with prior work linking emotional dysregulation to gambling severity and maladaptive decision-making in GD [9]. Consistent with this, recent gambling severity was itself associated with lower loss aversion, suggesting that reduced sensitivity to losses tracks not only emotional dysregulation but also the severity of recent gambling.

Alexithymia showed a complementary pattern. Higher TAS-20 scores were associated with reduced sensitivity to both gains and losses in the LCT, suggesting that alexithymic individuals may process outcome valence less distinctly even under relatively deliberative conditions. This finding extends earlier work on alexithymia and ambiguity-based decision-making in pathological gamblers [14] and is consistent with the broader view that difficulty identifying and describing feelings may compromise the use of emotional information during evaluation and choice. Notably, alexithymia was also associated with recent gambling severity and with avoidant decision-making style, reinforcing its clinical relevance in GD.

The profile analysis further suggests that emotional vulnerability in GD is not exhausted by general distress alone. The higher-risk cluster differed from the lower-risk cluster not only in the variables used to derive the solution, but also in alexithymia and a more spontaneous decisional style, whereas avoidant style and overall psychopathological distress did not distinguish the profiles—although, notably, depressive symptoms specifically did, even in the absence of a difference in general distress. This pattern is broadly consistent with conceptual models in which a subgroup of gamblers uses gambling primarily as a dysfunctional form of affect regulation [31, 32], and aligns with meta-analytic evidence linking gambling to facets such as impulse-control difficulties and self-blame [33]. Within this interpretation, gambling may function within a maladaptive cycle in which emotional distress is associated with gambling, gambling co-occurs with interpersonal and financial consequences, and these consequences may in turn relate to distress and suicide risk.

The null contrasts are informative rather than incidental: difficulty describing feelings, externally oriented thinking, avoidant and rational decision-making, emotional inattention, overall psychological distress, and anxiety did not differentiate the profiles. The high-risk group is thus distinguished specifically by difficulty identifying emotions, most facets of emotion dysregulation, spontaneous decisional tendencies, and depressive rather than global symptom load. Separating replication from novelty: the links of emotional dysregulation and alexithymia with gambling severity, and of alexithymia with avoidant coping, replicate prior work [9, 11], whereas the empirically derived risk profile—which also separates on suicidal ideation—and the low-loss-aversion/gambling-severity pairing are the more novel, still exploratory, contributions.

The present findings could also help clarify the relationship between alexithymia and suicide risk in GD. Previous studies have shown that alexithymia and broader emotion-regulation difficulties are associated with suicidal ideation across clinical and general-population samples [34, 35], with recent work indicating that depressive symptoms often mediate this association [36, 37]. This suggests that alexithymic traits may relate to suicide risk both directly and indirectly via depressive symptoms. Because the present design is cross-sectional, our results cannot establish a causal or temporal pathway; they are, however, consistent with the possibility that alexithymic traits are associated with suicide risk, a relationship that may operate through poorer emotional signaling, reduced anticipation of consequences, and greater reliance on avoidant coping. These mechanisms are offered as hypotheses for prospective testing.

From a clinical standpoint, these results are most directly relevant to assessment: they support more systematic assessment of emotional-regulation difficulties, alexithymia, gambling severity, and suicidality in GD services. Regarding treatment, the present study did not evaluate any intervention, so the following is a hypothesis for future research rather than an evidence-based recommendation: for patients resembling the high-risk profile, standard cognitive-behavioral approaches might be usefully supplemented with components targeting emotion regulation and avoidance—for example, Dialectical Behavior Therapy (DBT)-informed skills training focused on distress tolerance, emotional identification, and adaptive coping. Whether such components improve outcomes in GD, and specifically whether they reduce suicide risk, remains an open empirical question.

At the same time, the findings should be interpreted conservatively. The sample was modest, several analyses involved reduced valid n values, and the correlational and clustering approaches were explicitly exploratory. The data therefore support a preliminary, clinically suggestive pattern rather than a definitive mechanistic account.

Limitations and Future Directions: Several limitations must be acknowledged. The sample was small (n = 44; valid n as low as 21–24 for some analyses), limiting power and estimate stability—a sensitivity analysis found only correlations of |r| ≥ 0.41–0.58 and between-profile differences of d ≥ 1.0 reliably detectable, so null findings should not be read as evidence of no effect. The cross-sectional design precludes causal inference, and the predominantly male sample (93.2%) limits generalizability to women with GD, among whom distinct emotional profiles have been documented [38]. Suicidal ideation was dichotomized for clustering, reducing C-SSRS granularity, and the TAS-20 Difficulty Describing Feelings subscale had poor reliability (α = 0.456), as did—to a lesser degree—the GDMS Rational subscale (α = 0.66), warranting caution wherever they appear. Correlational findings remain exploratory despite FDR correction, confidence intervals, and Spearman checks; the cluster solution (n = 35) was reasonably stable under bootstrap resampling (adjusted Rand index = 0.73) but sensitive to standardization, and would need larger, confirmatory samples (e.g., latent profile analysis) for a definitive typology. Finally, although patients with documented dual pathology were excluded at intake, comorbidity was not otherwise systematically assessed or controlled, so the associations cannot be assumed specific to GD—medication status (31.8%) is the only available proxy, and because the exclusion targeted a formal diagnosis rather than medication use, this rate more plausibly reflects treatment of subclinical or GD-related symptoms than undisclosed comorbidity, though this cannot be verified without structured diagnostic data.

Future research should investigate the neural and psychophysiological correlates of the identified profiles using task-based functional imaging or other indices relevant to emotion-related decision-making in GD. Longitudinal studies are also needed to determine whether emotional dysregulation prospectively predicts suicidal ideation and gambling escalation, and to examine whether emotion regulation interventions can modify suicide risk trajectories in high-risk GD profiles.

## Conclusions

In summary, this study provides preliminary evidence that emotional dysregulation, alexithymia, maladaptive decisional tendencies, and suicidal ideation cluster meaningfully in a subgroup of individuals with GD. Although exploratory, the findings support a person-centered view of GD in which emotional vulnerability is closely tied to both decisional functioning and clinical severity. Further work is needed to replicate these profiles and determine their prognostic and treatment implications, but the present results suggest that emotion-focused and suicide-informed assessment should occupy a central place in the clinical evaluation of GD.

## Availability of Data and Materials

The data that support the findings of this study are available from the corresponding author upon reasonable request. The analysis code used to build the clean dataset and reproduce all statistics, tables, and figures reported here is openly available at https://doi.org/10.5281/zenodo.21470432. Raw participant-level data, including item-level questionnaire responses, are not publicly available to protect participant confidentiality—this is a small clinical sample (n = 44) that includes suicidality measures—but are available from the corresponding author on reasonable request, subject to the authors’ institutional and ethical approval.
